# *SBSPON* gene association with type 2 diabetes mellitus and its impact on leukocyte telomere length

**DOI:** 10.3389/fendo.2026.1799481

**Published:** 2026-04-16

**Authors:** Itty Sethi, Gh. Rasool Bhat, Ekta Rai, Swarkar Sharma, Suman Kotwal, Ankit Mahajan, Parvinder Kumar, Manoj K. Dhar

**Affiliations:** 1 Institute of Human Genetics, University of Jammu, Jammu, India; 2 Advanced Centre for Human Genetics, Sher.i.kashmir Institute of Medical Sciences (SKIMS), Srinagar, Jammu and Kashmir, India; 3 School of Life Sciences, Jawaharlal Nehru University, New Delhi, India; 4 Department of Zoology, Central University of Jammu, Jammu, India; 5 Department of Endocrinology, Government Medical College and Hospital, Jammu, India; 6 School of Biotechnology, University of Jammu, Jammu, India; 7 Department of Zoology, University of Jammu, Jammu, India

**Keywords:** genotyping, rs2291219, *SBSPON* gene, telomere length attrition, *TERF1*, type 2 diabetes mellitus (T2DM)

## Abstract

**Background:**

The study for the first time evaluates the association of the Somatomedin B and Thrombospondin Type 1 Domain Containing (*SBSPON*) gene with Type 2 Diabetes Mellitus (T2DM) in the Northwest Indian population. The present study investigates the expression profile of the gene, the association of variant rs2291219 with T2DM and its potential functional impact and relationship with leukocyte telomere length.

**Methods:**

The study is a case-control candidate gene association. The association of the *SBSPON* gene with T2DM risk was assessed using Gene expression, TaqMan genotyping, and *in silico* methods were employed to explore the potential functional effects of the variant. Additionally, the relationship between T2DM and leukocyte telomere length attrition was also examined.

**Results:**

Individuals with T2DM exhibited significantly higher expression levels compared to healthy controls. rs2291219 showed a significant association with T2DM (*p* = 0.02, OR 1.48, 95% CI: 1.06-2.07). The study indicates a higher risk among younger individuals. *In silico* analyses suggested a functional role for the variant. Individuals with T2DM who carried the risk allele showed shorter telomere length compared to healthy individuals (*p* = 0.004).

**Conclusion:**

This study provides evidence of *SBSPON* gene association with an increased risk of T2DM along with telomere length attrition. It could lay the groundwork for future research studies in a larger cohort. A potential role of *SBSPON* in the etiology of T2DM highlights the need for evaluation of genes in metabolic and other pathways apart from insulin.

## 1 Introduction

Type 2 Diabetes (T2DM) is a common progressive metabolic complex disorder, affecting 74.2 million individuals (20–79 years) in India (1). Worldwide, 1 in 8 adults is at high risk of developing T2DM. Significant studies have suggested that the genetic architecture of T2DM is polygenic (2–5). Genome-wide association studies (GWAS) have explained ~20% heritability (3, 6), mainly attributed to the genetic factors involved in the insulin pathways. The unexplained heritability of the disease remains debatable. Consequently, the need to explore other signaling pathways and genetic factors (variants and loci) that contribute to the pathogenesis of T2DM is a prerequisite (3, 7, 8). An important factor, recently found to be associated with T2DM, is Somatomedin B and Thrombospondin Type 1 Domain Containing (*SBSPON*) gene (9).

SBSPON, located on Chromosome 8q21.11, is comprised of 61,012 bases and encodes a protein of 264 amino acids. The gene of the thrombospondin (TSP) family is believed to play a very important role in the metabolism and O-glycosylation pathways. mRNA expression in normal human tissues from the GTEx and cBio portal data showed that the gene is expressed in several tissues, including whole blood, brain, heart, skeletal muscle, adipocyte, kidney, liver, lung, pancreas and thyroid (10). The available data from various databanks suggested a secretory role of the gene, while the protein expression analysis revealed the expression in the exocrine glandular cells of the pancreas. It can be hypothesized that any mutation in the gene or abnormal function of the gene can lead to pancreatitis and diabetes. The gene showed moderate expression in pancreas (alpha, beta and islets) tissue and PANC-1 cell lines (11). The variants (rs2291219 and rs2291220) in the gene have been observed to be related to T2DM and telomere length attrition in the Caucasian Female population (9, 12). The variants were linked with the Telomere Repeat Binding Factor 1 (TERF1) gene; however, these are located in exons 4 and 5 of the SBSPON gene, respectively. Therefore, it is not specified how the gene plays playing role in T2DM etiology. It might be possible that the gene is also involved in telomere length regulation (12). Studies have explored the Indian population and showed a significant association of telomere length attrition with T2DM individuals (13–15). To date, no perspective data is available on the SBSPON gene. However, by analyzing the available data, it can be anticipated that the gene plays an important role in physiological pathways. Therefore, there is a need to determine the role of this gene in T2DM etiology and screening of the Indian population for the genetic variations in the gene in association with T2DM to gain more insight into the pathophysiology of the disease.

In the present study, to elucidate the role of gene we analyzed the expression of the gene, the association of the variant rs2291219 in *SBSPON* with T2DM in the population of Northwest India and the association of telomere length attrition and T2DM.

## 2 Materials and methods

### 2.1 Ethical statement

Ethical approval has been obtained from the Institutional Ethical Committee, University of Jammu, under notification no. DRS/22/4961 and Government Medical College and Hospital, Jammu, under notification no. IEC/GMCJ/2024/1691. The study was performed in accordance with the relevant guidelines and regulations, with written informed consent from all the participants enrolled.

### 2.2 Subjects

A total of 1000 randomly collected samples were recruited to explore the variant and leukocyte telomere length attrition in the present study, which includes 500 individuals with diabetes and 500 healthy individuals belonging to the Northwest Indian population. An independent cohort of 100 samples were recruited for the gene expression analysis. A diagnostic criterion was made according to the International Diabetes Federation (IDF). Epidemiological characteristics are summarized in **Table 1**. The control group has postprandial glucose levels below 11mmol/L, fasting glucose levels below 7mmol/L, HbA1c < 5.7% with no family history of T2DM.

**Table 1:** Distribution of epidemiological parameters between T2DM individuals and Healthy individuals from the population of Northwest India.

S.No.	Characteristic	Cases* (mean ± SD)	Controls (mean ± SD)	*P*-value	Bonferroni-adjusted *p*-value
1	Age in years (min-max)	(24–86) 51.7 ± 10.08	(21–86) 53.83 ± 11.88	0.003	0.033
2	Gender – Male (%)	41.9%	51.2%	–	–
	Gender – Female (%)	57.9%	48.6%	–	–
3	BMI-kg/m2	26.2 ± 4.7	25.13 ± 3.7	6.09 × 10^-5^	6.70 × 10^-4^
4	Systolic Blood Pressure (SBP)-mmHg	135.76 ± 13.38	126.72 ± 13.5	5.05 × 10^-^¹³	5.56 × 10^-^¹²
5	Diastolic Blood Pressure (DBP)-mmHg	83.71 ± 7.13	81.97 ± 6.43	0.002	0.022
6	Random Blood Glucose -mg/dl	226.76 ± 70.5	119.19 ± 20.22	6.79 × 10^-77^	7.47 × 10^-76^
7	Fasting Blood Glucose -mg/dl	156.28 ± 57.6	83.43 ± 8.68	9.85 × 10^-46^	1.08 × 10^-44^
8	Triglycerides	215.39 ± 123.65	163.6 ± 72.8	1.78 × 10^-^¹²	1.96 × 10^-^¹¹
9	High-Density Lipoproteins (HDL)	46.33 ± 14.9	46.6 ± 14.66	0.7	1.000
10	Very Low-Density Lipoprotein (VLDL)	43.41 ± 27.5	33.03 ± 15.25	1.2 × 10^-^¹^0^	1.32 × 10^-9^
11	Cholesterol	172.02 ± 40.26	160.81 ± 42.7	0.0004	0.0044
12	Low-Density Lipoproteins (LDL)	82.87 ± 29.49	81.92 ± 40.36	0.9	1.000

* Other clinical conditions removed.

### 2.3 Gene expression

For Gene expression, an independent cohort of 100 random samples were recruited from the population of Jammu and Kashmir, Northwest India. The total RNA was isolated from whole blood using RNA extraction kit (Genes2me, India). RNA integrity and purity were confirmed by 1.5% agarose gel electrophoresis and O.D. 260/280 absorbance ratio of 1.9, respectively. Before cDNA synthesis, RNA was treated with DNase I (Puregene, Genetix Biotech). The cDNA was prepared using the high-fidelity cDNA synthesis kit (Genes2me, India), as per the manufacturer’s instructions. The expression was measured by SyBr Green chemistry on Biorad CFX384 Touch Real-Time System (Bio-Rad Laboratories, Inc.). Primers for *SBSPON* and reference gene (scg) *ALBUMIN* were supplied by IDT (**Supplementary Table 1**). The qPCR conditions used were Stage 1: single cycle of 95°C for 5 minutes; Stage 2: 40 cycles of 95°C for 10 seconds, 62°C for 15 seconds, 72°C for 1 minute with the signal acquisition for GOI and SCG product; Stage 3: a single default cycle (Bio-rad CFX 1000 Touch Real-Time system) of dissociation curve – 95°C for 10 seconds, 65°C - 95°C at 0.5 °C increment for 5 seconds. The final concentration of reagents in each of 5 µl reaction mixture were: 1X SYBR Green Master mix (Genes2me, India), 0.5µM Forward primer, 0.5µM Reverse primer, 0.5 µM albu_F primer, 0.5 µM albd_R primer (IDT), and ~10ng cDNA. The data was exported in excel format from the CFX BioRad Software and analyzed by the Ct method (16) further, logistic regression was applied by IBM SPSS Statistics v.20 software (17). The cross validation was conducted by running 25 random samples. Two no template controls (NTC) were also included in each run. All samples were processed under identical laboratory conditions, thereby reducing potential technical variability and minimizing the likelihood of batch-related artifacts.

### 2.4 Genotyping

In the present study, DNA was isolated from the collected blood samples using phenol/chloroform: isoamyl alcohol methodology. Qualitative and quantitative analyses were performed on the isolated blood samples. Variant rs2291219 of the *SBSPON* gene was genotyped using TaqMan allele discrimination assay on Biorad CFX384 Touch Real-Time System (Bio-Rad Laboratories, Inc.). Taqman^®^ SNP genotyping assays (Primer and Probe labeled with Fam and VIC) supplied by Applied Biosystems (Thermo Fischer Scientific, Pleasanton, CA, USA) and Taqman^®^ master mix II with UNG supplied by Applied Biosystems (Thermo Fischer Scientific, Foster City, CA, USA). The genotyping was performed by following the recommended master mix concentration and PCR conditions. The BioRad CFX384 Touch Real-Time System was used to measure allele-specific fluorescence. All samples were run in 384-well plate formats. Samples failing genotyping or with ambiguously called genotypes were repeated for final inclusion. The cross-validation was conducted by picking 100 random samples and re-genotyping. Two no template controls (NTC) were also included in each set to keep a check on extraneous nucleic acid contamination.

### 2.5 Telomere length measurement

Leukocyte Telomere Length was assessed by utilizing dual-labeled fluorescence probe-based assay (18). Literature survey revealed that the variant is associated with the telomere maintenance gene, *TERF1* (9). Therefore, in the present study, the telomere length was analyzed (n = 310) in both the cases and controls. The data was exported in Excel format from the CFX BioRad Software. The samples were run in duplicates, and then the averaged Ct (Threshold) value was used to calculate relative telomere length (RTL). RTL was calculated based on Ct values, as [2Ct (telomeres)/2Ct (scg)]–1 = 2–ΔCt (16).

### 2.6 Statistical analyses

Clinical characteristics were compared between cases and controls using a t-test (**Table 1**). Statistical analyses on the genotype data were performed by using IBM SPSS Statistics v.20 software (17). The association of variant with T2DM was confirmed by binary logistic regression adjusted for confounding factors - age, gender and Body Mass Index (BMI). The odds ratios (ORs) were calculated with respect to the risk allele found in this study (**Table 2**). Correlation of variant with the characteristics of the disease was performed by Pearson Correlation analysis in the whole population and on the basis of gender (**Supplementary Tables S2**, **S3**). The comparison of clinical characteristics of different genotypes for the variant was performed by one-way ANOVA, adjusted for age and gender (**Supplementary Tables S4**, **S5**).

**Table 2:** Allelic distribution.

Variant	rs2291219		
GENE	*SBSPON*		
POLYMORPHISM (ref/alt-GRCh38/hg38)	A/G		
ANCESTRAL ALLELE	G		
ALLELE DISTRIBUTION	A		G
CASES	0.47		0.53
CONTROLS	0.41		0.59
ALLELIC ODDS RATIO at 95% CI	1.30 (1.03-1.57)		
RISK ALLELE	A		
GENOTYPIC MODEL	Recessive (AA vs GG+AG)		
*p*-VALUE	Unadjusted	Adjusted	
	0.01	0.02*	
ODDS RATIO at 95% CI	Unadjusted	Adjusted	
	1.5 (1.09-2.11)	1.48 (1.06-2.07) *	
H.W.E (Cases/Controls/Total)	0.1/0.5/0.05^#^		

*Adjusted for Age, Gender, and BMI; ^#^ The borderline values may in total population may arise due to sampling variation, modest sample size, or population substructure/heterogeneity and do not necessarily indicate a true deviation from equilibrium.

### 2.7 Putative functional role of the variant

The combined expression Quantitative Trait Loci (eQTL) analysis of the variant was explored by using the University of California Santa Cruz (UCSC) Genome Browser (https://genome.ucsc.edu) and the GTEx portal (https://www.gtexportal.org). The transcriptional regulatory roles like histone modifications, DNase hypersensitivity and binding sites for the transcription factor, were analyzed by UCSC Genome Browser, Encyclopedia of DNA Elements (ENCODE) (V3) and Haploreg v4.1 database. The effect of the variant on splicing by using the web tool Human Splicing Finder (HSF) 3.1 and ESE Finder (3.0) was also analyzed (**Table 3**). The protein structure was analyzed by exploring the i-Tasser software (19, 20).

**Table 3:** Putative Role of the variant with T2DM in the studied population (Jammu and Kashmir, Northwest India) utilizing the information from the various databases, including GTEX, UCSC genome browser, ENCODE, Haploreg and HSF.

Variant	Location of the variant w.r.t nearest gene	Allele ref/alt (risk allele)*	eQTL gene	eQTL tissue	eQTL sample size	eQTL NES	eQTL p-value	eQTL m-value	Putative role (cis- eQTL) of variant	Direction of Effect (risk allele A)	Regulatory role of variant (encode and haploreg data)	Splicing effect
rs2291219	4^th^ exon of *SBSPON* and Downstream to *TERF1* (missense)	A/G (A)*	*SBSPON*	Pancreas	305	-0.331	**1.9E-6**	1	**Significant and downregulation**	↓ Expression	H3K27ac_Enh, DNase; potentially alters five regulatory motifs, ATF3_disc3, HNF4_known1, HNF4_known8, NF-AT, and ZBTB7A_disc2.	New site for SF2/ASF-76.70
				Adipose-subcutaneous	581	-0.033	0.1	0.08	Non-Significant	Slight ↓ expression		
				Skeletal Muscle	706	-0.012	0.7	0.05	Non-Significant	No clear effect		
				Whole Blood	670	-0.149	**7.7E-4**	1	**Significant and downregulation**	↓ Expression		
			*TERF1*	Pancreas	305	-0.306	**1.2E-9**	1	**Significant and downregulation**	↓ Expression		
				Adipose-subcutaneous	581	0.0096	0.7	0	Non-Significant	Slight ↑ expression		
				Skeletal Muscle	706	0.05	0.08	0	Non-Significant	Slight ↑ expression		
				Whole Blood	670	0.003	0.9	0	Non-Significant	No clear effect		

* - Risk Allele; ↓ - decreased expression; ↑ - increased expression.

## 3 Results

### 3.1 Clinical parameters of the population

The clinical parameters of the population studied are mentioned in **Table 1**. It demonstrated that population had larger number of T2DM females. Significant differences had been detected in BMI, systolic and diastolic blood pressure, blood glucose, triglycerides, very low density lipo-proteins and cholesterol (p-value < 0.001). The parameters remain statistically significant even after Bonferroni correction (p-value < 0.05).

### 3.2 Gene expression analysis

A total of independent random 100 samples were employed for the gene expression analysis. A significant difference of expression was observed among the cases and controls. T2DM individuals showed 2 times elevated levels of expression as compared to the healthy individuals with p-value of 0.02 adjusted for age and gender (**Supplementary Figure 1**). Further, Receiver Operating Characteristic (ROC) curve yielded an AUC of 0.71 with 95% CI indicating a moderate predictive accuracy.

### 3.3 Association of variant rs2291219 with T2DM

The association of variant rs2291219 with T2DM was explored in the population of Northwest India. The variant was observed to be significantly associated with T2DM. In the present study, the minor allele A showed the risk for T2DM with a p-value of 0.02 adjusted for age, gender and BMI with an odds ratio of 1.48 (1.06-2.07). The variant follows the recessive model (AA vs GG+AG), and the population was in Hardy-Weinberg equilibrium (**Table 2**). Correlation analysis revealed nominally significant association of age and age of onset with T2DM in males and overall population respectively however, did not remain significant after Benjamini–Hochberg FDR correction. One-way ANOVA revealed significant genotype-associated differences with lipid parameters, which remained significant after FDR correction (pFDR = 0.037) (**Supplementary Tables S2**–**S5**).

### 3.4 Leukocyte telomere length attrition in T2DM individuals

Studies showed the variant is associated with the telomere maintenance genes (9, 12). Further, supported by the studies reporting telomere shortening in T2DM individuals in Indian population (13, 21). Therefore, the telomere length in the T2DM individuals was examined in comparison to the healthy controls. It was observed that there is a significant difference in telomere length among the cases carrying risk genotype (AA) and controls with a p-value of 0.004 adjusted for age, gender and BMI (**Table 4**; **Supplementary Table 6**).

**Table 4:** Binary logistic regression analysis of leukocyte telomere length (LTL) among the individuals with T2DM and healthy individuals having the risk allele (A) observed in the studied population.

LTL	Beta coefficient	*P*-value	VIF
Unadjusted	-0.43	**0.01**	
Adjusted for			
Age, Gender and BMI	-0.512	**0.004**	
Age, Gender, BMI, and FBS	-1.2	**0.007**	
LTL			1.09
Age			1.06
Gender			1.06
BMI			1.07
FBS			1.06

Bold values indicate a statistically significant association between leukocyte telomere length (LTL) and type 2 diabetes mellitus (T2DM) (p < 0.05).

### 3.5 *In silico* analyses

The putative functional role of the variant rs2291219 was analyzed by the GTEx database. It was observed that the variant significantly downregulated the expression of the SBSPON gene in pancreas (p-value = 1.9E-6) and whole blood (p-value = 7.7E-4). The GTEx database showed that the variant also regulated the expression of another gene, which is responsible for the maintenance and protection of telomeres, TERF1 (p-value = 1.2E-9). The variant significantly downregulated the expression of the TERF1 gene in the pancreas. GTEx data revealed that the gene is also overexpressed in the Nerve - Tibial (x6.6), Artery - Coronary (x4.8), Artery - Tibial (x4.5), and Esophagus - Gastroesophageal Junction (x4.2) (22). The variant also acts as a sQTL. Exon Splicing Enhancer Finder 3.0 database showed the changes in binding sites for SR proteins when both alleles were analyzed (**Supplementary Figure 2**) (23). The EMBL-IntAct database showed the interaction of the SBSPON protein with proteins GFER, ZNF609 and ZNF703 (**Supplementary Figure 3**). The variant also has multiple SNPs in the TERF1 gene in strong linkage with it. The region showed the presence of the Histone marker H3K27ac_Enh, H3K4me1_Panc1_hg19_1, H3K4me2_skeletal muscle myoblast_hg19_1, H3K4me3_brown adipose tissue_mm9_1, H3K4me3_skeletal muscle cell_hg19_1 and DNase. Interestingly, the HaploReg data showed that the variant potentially alters five regulatory motifs, ATF3_disc3, HNF4_known1, HNF4_known8, NF-AT, and ZBTB7A_disc2 (**Table 3**).

Both the protein sequences (wild type and mutant type) for rs2291219, with arginine as an amino acid in one model and tryptophan in the other model of the protein, were analyzed by using i-Tasser and i-Tasser MTD, a bioinformatics tool (19, 20). Analysis of the generated protein structures identified differences in domain composition. The variant containing tryptophan (G allele) exhibited three domains, while the variant with arginine (A allele) displayed four domains (**Supplementary Figure 4**). Ligand binding site analysis indicated altered ligand interactions at the same site for both variants (**Supplementary Figures S5**–**S7**). Structural examination demonstrated dynamic conformational changes associated with each amino acid substitution (**Supplementary Figure 8**).

## 4 Discussion

The study provides the first evidence from the Northwest India population that elucidates the significant role of the SBSPON gene in Type 2 Diabetes Mellitus (T2DM) pathogenesis. The increasing complexity and prevalence of the disease necessitate an expanded exploration of alternative genetic pathways and loci contributing to disease susceptibility. Additionally, it was reported in few studies (24–26) that thrombospondin 1 (TSP1) has high expressivity in obese, insulin resistant with high adipose inflammation and Diabetes kidney disease individuals and contributes in pathological processes like insulin resistance and adipose tissue inflammation. Both SBSPON and TSP-1 drive their biological functions via the conserved Thrombospondin-1 domain. SBSPON may have overlapping functions in metabolic and inflammatory pathways which suggests a potential role in the pathogenesis of T2DM by regulating metabolism, inflammatory responses and oxidative stress. Therefore, the present study has diverged from the conventional research focus on insulin pathway-related genetic factors in T2DM, and examined the relatively understudied SBSPON gene, its variant rs2291219 and the impact of the risk allele on leukocyte telomere length.

The study showed elevated levels of gene expression among the T2DM individuals. The expression level correlates with Human Protein Atlas data where the SBSPON protein showed elevated levels in blood. Further, in the pan-disease Olink HT protein profiling dataset, the T2DM cohort demonstrates a moderate shift in the NPX distribution suggesting the modest alteration in the protein profile may serve as early predictors of disease progression (27). Although, the AUC of 0.71 indicates the moderated prediction accuracy however, the gene may serve as biomarker for multi-marker panel studies. It aligns with the early phase single biomarker discovery studies expectations where the AUC ≥ 0.7 is acceptable to take the biomarker for further evaluation (28). Despite providing supportive functional evidence for disease etiology, the present expression study is limited by sample size. Therefore, validation in larger cohorts considering genetic heterogeneity is necessary in future studies.

In the present study, the observed allele frequencies in controls (A = 0.40; G = 0.59) is similar to the South Asian distribution (A = 0.39; G = 0.60), supporting the representativeness of the studied population. Further, the global allele frequencies (A = 0.314; G = 0.69) also provide an important reference for comparison. In contrast, T2DM cases exhibited a relatively higher frequency of the A allele (A = 0.47; G = 0.53), suggesting a possible enrichment of the A allele among affected individuals in the study population. This shift in allelic distribution may indicate a potential association of the A allele with disease susceptibility; however, further studies with larger sample sizes and diverse populations are required to validate this observation. While analyzing the clinical parameters, it was observed that age was associated with T2DM, and cases were younger than controls on average (a worrisome sign). The risk genotype showed a negative Pearson correlation with age of onset, with a p-value of 0.04. Further, the gender analysis also showed that males have a negative correlation of age with risk genotype (p-value of 0.02). One-way ANOVA analysis also showed an association of age of onset with risk genotype (AA) (**Supplementary Tables S2**, **S5**). However, these associations did not remain statistically significant after controlling for multiple testing using the Benjamini–Hochberg false discovery rate (FDR) correction. This suggests that the observed correlations may represent potentially weak associations arising from multiple comparisons. Such weak correlations are common in complex multifactorial diseases like type 2 diabetes mellitus, where genetic, environmental, and metabolic factors collectively influence disease susceptibility and progression. Despite being nominal significant associations, the findings suggests that individuals are developing T2DM at an early age in the studied population. This is in reference to the reported studies that individuals are developing T2DM at early ages. Earlier onset of T2DM in young individuals (<40 years) can be ascribed to obesity, sedentary lifestyle and genetic changes. Individuals at a young age (<40 years) have increased insulin insensitivity and functional deterioration of beta cells. Young individuals are more prone to T2DM complications due to a longer duration of the disease (29–31). The variant showed the highest positive enrichment for BMI, Triglycerides and negative for HDL cholesterol in the common metabolic diseases knowledge portal (T2D KP) (32, 33). Interestingly, lipid traits, including cholesterol, triglycerides, and VLDL showed significant genotype-associated differences even after FDR correction, whereas glycemic traits showed no significant association. This may suggest that the studied variant potentially may influences the lipid metabolism rather than directly affecting glycemic regulation in individuals with type 2 diabetes.

The leukocyte telomere length was shorter among the cases carrying the risk allele than the controls. The negative beta coefficient suggested an inverse relationship, indicating increased telomere length attrition among T2DM individuals (**Table 4**). The leukocyte telomere length showed a significant inverse Pearson’s correlation with glycemic traits fasting and random blood sugar (**Supplementary Table 7**), indicating higher the sugar levels shorter the telomere length. However, the findings did not remain significant after FDR analysis. The findings suggest a potential association between the studied variant and telomere length. The variant was shown to be associated with the telomere maintenance gene, TERF1 and may be involved in the cis-regulation that correlates with the present study (9, 12).

The in-silico analyses intimates the involvement of SBSPON gene in the etiology of T2DM. The significant downregulation of the SBSPON expression observed in the pancreatic tissue from GTEx database emphasizes the role of the gene and its variant in pancreatic homeostasis and β-cell function. β-cells play a central role in glucose homeostasis and altered expression of the gene may contribute to the impaired signaling and regulation of insulin, a major hormone involved in the pathophysiology of T2DM. Considerably, rs2291219 also demonstrated a strong regulatory effect on TERF1, a critical and integral component of the shelterin complex responsible for telomere protection and maintenance (12). The downregulation of the TERF1 in the pancreas further suggests a link between the telomere maintenance genes and pancreatic cell dysfunction. Telomere length is regulated and maintained by the telomere maintenance genes and polymorphism in these genes has been reported to be associated with telomere length attrition in diabetes (12, 34). Telomere length attrition has been widely implicated with the T2DM contributors, oxidative stress, impaired glucose tolerance, obesity, cellular senescence, inflammation and insulin resistance (35–38). The simultaneous expression dysregulation of both the genes, SBSPON and TERF1 emphasizes the convergence involving extracellular matrix signaling and telomere genomic stability in the pathophysiology of T2DM.

The variant identifies as a splice quantitative trait locus (sQTL). The allele-specific alterations in SR protein binding sites may alter the protein stability, localization and interaction which can further amplify the biological effects of the variant in gene expression. Protein interaction analysis revealed that the SBSPON interacts with GFER, ZNF609, and ZNF703. The GFER is important for oxidative phosphorylation and mitochondrial maintenance. The ZNF609 is involved in modulating inflammation and insulin resistance in the adipose tissue (39) and ZNF703 is in the embryonic development and cell proliferation and found to be associated with T2D in GWAS of Japanese and East Asian population (40). Altered interaction of SBSPON-GFER could influence the mitochondrial function, an important regulatory mechanism of insulin resistance and β-cell failure (41, 42). Furthermore, the reported association of ZNF860 with T2DM in EWAS analysis supports the involvement of zinc-finger transcription factors in diabetes etiology (43). The presence of active regulatory elements, H3K27ac_Enh, H3K4me1_Panc1_hg19_1, H3K4me2_skeletal muscle myoblast_hg19_1, H3K4me3_brown adipose tissue_mm9_1, H3K4me3_skeletal muscle cell_hg19_1 and DNase hypersensitivity sites indicates the genomic region is transcriptionally active. The predicted alteration of key regulatory motifs, including HNF4 and NF-AT, is particularly noteworthy. HNF4 is a master regulator of pancreatic gene expression and glucose metabolism (44), while NF-AT signaling is involved in β-cell proliferation and insulin transcription (45). Disruption of these motifs may therefore result in profound metabolic consequences.

Structural modeling of the SBSPON protein revealed that the rs2291219 variant induces conformational and domain architectural changes, with the tryptophan-containing variant exhibiting fewer domains than the arginine-containing wild-type protein. Tryptophan, an aromatic amino acid, participates in glycan-protein interactions, whereas arginine is classified as a polar amino acid. The protein is implicated in glycosylation, and such structural alterations can significantly impair protein function, including ligand binding, protein–protein interactions, and intracellular signaling thereby affecting downstream metabolic pathways relevant to T2DM. Although the in-silico analyses provides important insights, these findings warrant further experimental validation. Further, it was observed in eQTL analysis that the risk allele of the variant is downregulating the gene expression however the study observed the elevated expression. This apparent difference between the eQTL findings and the observed expression levels may be attributed to the fact that eQTL analyses capture genotype-dependent regulatory effects, whereas gene expression studies measure overall transcript abundance within the sampled population. Therefore, the genotype/allele-specific effect on the gene expression should be evaluated in a larger sample size in future studies.

Overall, the study provides substantiate evidence suggesting a potential role of SBSPON gene in T2DM etiology. The integration of transcriptomic, genomic, epigenomic, and structural bioinformatics analyses strengthens the biological plausibility of this gene and its variant as a functional risk determinant for T2DM. The present study has limitations, including a sample size and possible genetic heterogeneity. Further studies from larger cohorts with different ethnic groups along with functional validation through in vitro and in vivo studies are requisite to confirm these computational predictions thereby elucidating the role of SBSPON in T2DM etiology.

## 5 Conclusion

The study demonstrates a significant elevated expression of the gene among the T2DM individuals, with moderate predictive accuracy of AUC = 0.71. The risk allele (A) carriers exhibit a 1.48-fold increased risk of having T2DM compared to the healthy individuals. The study reveals gender-specific patterns, particularly the inverse correlation between the risk allele and age of onset observed exclusively in males, suggesting potential sex-linked mechanisms in disease progression. This gender dimorphism emphasizes the necessity of personalized medicine strategies in T2DM and supports the implementation of sex-specific therapeutic interventions. In silico analyses provide mechanistic insights into the variant’s functional significance, suggesting the variant’s biological relevance in T2DM pathophysiology. The association of variant with telomere maintenance genes makes it an ideal candidate to study the telomere length. The significant reduction in telomere length observed in T2DM patients harboring the risk allele, even after adjusting for demographic and anthropometric confounders, establishes telomere attrition as an independent predictor of disease susceptibility. The inverse correlation between blood glucose levels and telomere length further supports the interconnected relationship between metabolic dysfunction and cellular aging mechanisms. The findings offer the preliminary evidence for the gene’s potential role as biomarker for T2DM risk. Further studies in larger, independent and homogenous cohort along with exploration of the underlying mechanistic pathway and functional experimental validation of the in-silico analyses could delineate the role of the gene in T2DM etiology. The identification of SBSPON as a potential therapeutic target can open new avenues for drug development and personalized treatment strategies.

## Data Availability

The original contributions presented in the study are included in the article/**Supplementary Material**. Further inquiries can be directed to the corresponding authors.

## References

[1] Sun H Saeedi P Karuranga S Pinkepank M Ogurtsova K Duncan BB . Idf diabetes atlas: global, regional and country-level diabetes prevalence estimates for 2021 and projections for 2045. Diabetes Res Clin Pract. (2022) 183:109119. doi: 10.1016/j.diabres.2021.109119. PMID: 34879977 PMC11057359

[2] Kolb H Martin S . Environmental/lifestyle factors in the pathogenesis and prevention of type 2 diabetes. BMC Med. (2017) 15:131. doi: 10.1186/s12916-017-0901-x. PMID: 28720102 PMC5516328

[3] Morris AP . Progress in defining the genetic contribution to type 2 diabetes susceptibility. Curr Opin Genet Dev. (2018) 50:41–51. doi: 10.1016/j.gde.2018.02.003. PMID: 29477131

[4] Prasad RB Groop L . Genetics of type 2 diabetes-pitfalls and possibilities. Genes. (2015) 6:87–123. doi: 10.3390/genes6010087. PMID: 25774817 PMC4377835

[5] Kaprio J Tuomilehto J Koskenvuo M Romanov K Reunanen A Eriksson J . Concordance for type 1 (insulin-dependent) and type 2 (non-insulin-dependent) diabetes mellitus in a population-based cohort of twins in Finland. Diabetologia. (1992) 35:1060–7. doi: 10.1007/bf02221682. PMID: 1473616

[6] Xue A Wu Y Zhu Z Zhang F Kemper KE Zheng Z . Genome-wide association analyses identify 143 risk variants and putative regulatory mechanisms for type 2 diabetes. Nat Commun. (2018) 9:2941. doi: 10.1038/s41467-018-04951-w. PMID: 30054458 PMC6063971

[7] Fuchsberger C Flannick J Teslovich TM Mahajan A Agarwala V Gaulton KJ . The genetic architecture of type 2 diabetes. Nature. (2016) 536:41–7. doi: 10.1038/nature18642. PMID: 27398621 PMC5034897

[8] Scott RA Scott LJ Magi R Marullo L Gaulton KJ Kaakinen M . An expanded genome-wide association study of type 2 diabetes in Europeans. Diabetes. (2017) 66:2888–902. doi: 10.2337/db16-1253. PMID: 28566273 PMC5652602

[9] Zee RYL Ridker PM Chasman DI . Genetic variants of 11 telomere-pathway gene loci and the risk of incident type 2 diabetes mellitus: the women’s genome health study. Atherosclerosis. (2011) 218:144–6. doi: 10.1016/j.atherosclerosis.2011.05.013. PMID: 21665207 PMC3175791

[10] Gtex Portal . (2025). Available online at: https://www.gtexportal.org/home/gene/SBSPON (Accessed January 02, 2025).

[11] Lachmann A Torre D Keenan AB Jagodnik KM Lee HJ Wang L . Massive mining of publicly available rna-seq data from human and mouse. Nat Commun. (2018) 9:1366. doi: 10.1038/s41467-018-03751-6. PMID: 29636450 PMC5893633

[12] Sethi I Bhat GR Singh V Kumar R Bhanwer AJ Bamezai RN . Role of telomeres and associated maintenance genes in type 2 diabetes mellitus: a review. Diabetes Res Clin Pract. (2016) 122:92–100. doi: 10.1016/j.diabres.2016.10.015. PMID: 27816684

[13] Adaikalakoteswari A Balasubramanyam M Mohan V . Telomere shortening occurs in Asian Indian type 2 diabetic patients. Diabetic Medicine: A J Br Diabetic Assoc. (2005) 22:1151–6. doi: 10.1111/j.1464-5491.2005.01574.x. PMID: 16108841

[14] Rai S Badarinath ARS George A Sitaraman S Bronson SC Anandt S . Association of telomere length with diabetes mellitus and idiopathic dilated cardiomyopathy in a South Indian population: a pilot study. Mutat Res Genet Toxicol Environ Mutagen. (2022) 874-875:503439. doi: 10.1016/j.mrgentox.2021.503439. PMID: 35151422

[15] Piplani S Alemao NN Prabhu M Ambar S Chugh Y Chugh SK . Correlation of the telomere length with type 2 diabetes mellitus in patients with ischemic heart disease. Indian Heart J. (2018) 70:S173–6. doi: 10.1016/j.ihj.2018.09.007. PMID: 30595252 PMC6310747

[16] Schmittgen TD Livak KJ . Analyzing real-time pcr data by the comparative c(t) method. Nat Protoc. (2008) 3:1101–8. doi: 10.1038/nprot.2008.73. PMID: 18546601

[17] IBM Corp . IBM SPSS Statistics for Windows, Version 20.0. Armonk, NY: IBM Corp. (2011).

[18] Sethi I Bhat GR Kumar R Rai E Sharma S . Dual labeled fluorescence probe based qpcr assay to measure the telomere length. Gene. (2021) 767:145178. doi: 10.1016/j.gene.2020.145178. PMID: 33007378

[19] Zheng W Wuyun Q Li Y Liu Q Zhou X Peng C . Deep-learning-based single-domain and multidomain protein structure prediction with D-I-Tasser. Nat Biotechnol. (2025). doi: 10.1038/s41587-025-02654-4. PMID: 40410405 PMC13090124

[20] Zhou X Zheng W Li Y Pearce R Zhang C Bell EW . I-Tasser-Mtd: a deep-learning-based platform for multi-domain protein structure and function prediction. Nat Protoc. (2022) 17:2326–53. doi: 10.1038/s41596-022-00728-0. PMID: 35931779

[21] Sethi I Sharma V Sharma I Singh G Bhat GR Bhanwer AJS . Telomere maintenance genes are associated with type 2 diabetes susceptibility in northwest Indian population group. Sci Rep. (2020) 10:6444. doi: 10.1038/s41598-020-63510-w. PMID: 32296102 PMC7160122

[22] Gtex Portal_Variant . (2025). Available online at: https://www.gtexportal.org/home/snp/rs2291219 (Accessed January 02, 2025).

[23] Cartegni L Wang J Zhu Z Zhang MQ Krainer AR . Esefinder: a web resource to identify exonic splicing enhancers. Nucleic Acids Res. (2003) 31:3568–71. doi: 10.1093/nar/gkg616. PMID: 12824367 PMC169022

[24] Varma V Yao-Borengasser A Bodles AM Rasouli N Phanavanh B Nolen GT . Thrombospondin-1 is an adipokine associated with obesity, adipose inflammation, and insulin resistance. Diabetes. (2008) 57:432–9. doi: 10.2337/db07-0840. PMID: 18057090 PMC2877915

[25] Matsuo Y Tanaka M Yamakage H Sasaki Y Muranaka K Hata H . Thrombospondin 1 as a novel biological marker of obesity and metabolic syndrome. Metab Clin Exp. (2015) 64:1490–9. doi: 10.1016/j.metabol.2015.07.016. PMID: 26298466 PMC4936918

[26] Liu Z Qin Z Bai W Wang S Huang C Li N . Integrating bioinformatics and machine learning to elucidate the role of protein glycosylation-related genes in the pathogenesis of diabetic kidney disease. PloS One. (2025) 20:e0329640. doi: 10.1371/journal.pone.0329640. PMID: 40825025 PMC12360563

[27] The Human Protein Atlas . Available online at: https://www.proteinatlas.org/ENSG00000164764-SBSPON/blood#pan_disease_ht_pea (Accessed January 03, 2025).

[28] Rifai N Gillette MA Carr SA . Protein biomarker discovery and validation: the long and uncertain path to clinical utility. Nat Biotechnol. (2006) 24:971–83. doi: 10.1038/nbt1235. PMID: 16900146

[29] Rodriquez IM O'Sullivan KL . Youth-onset type 2 diabetes: burden of complications and socioeconomic cost. Curr Diabetes Rep. (2023) 23:59–67. doi: 10.1007/s11892-023-01501-7. PMID: 36961664 PMC10037371

[30] Magliano DJ Sacre JW Harding JL Gregg EW Zimmet PZ Shaw JE . Young-onset type 2 diabetes mellitus — implications for morbidity and mortality. Nat Rev Endocrinol. (2020) 16:321–31. doi: 10.1038/s41574-020-0334-z. PMID: 32203408

[31] Viner R White B Christie D . Type 2 diabetes in adolescents: a severe phenotype posing major clinical challenges and public health burden. Lancet (London England). (2017) 389:2252–60. doi: 10.1016/s0140-6736(17)31371-5. PMID: 28589895

[32] Costanzo MC von Grotthuss M Massung J Jang D Caulkins L Koesterer R . The type 2 diabetes knowledge portal: an open access genetic resource dedicated to type 2 diabetes and related traits. Cell Metab. (2023) 35:695–710.e6. doi: 10.1016/j.cmet.2023.03.001. PMID: 36963395 PMC10231654

[33] Type 2 Diabetes Knowledge Portal (T2dkp) . (2025). Available online at: https://t2d.hugeamp.org/variant.html?variant=8%3A73982161%3AA%3AG (Accessed January 03, 2025).

[34] Zhu H Belcher M van der Harst P . Healthy aging and disease: role for telomere biology? Clin Sci (London England: 1979). (2011) 120:427–40. doi: 10.1042/cs20100385. PMID: 21271986 PMC3035527

[35] Baliou S Apetroaei M-M Hatzidaki E Kuzmin SV Tzatzarakis MN Arsene AL . The interplay between obesity and type 2 diabetes: common pathophysiological mechanisms contributing to telomere shortening. Life. (2025) 15:873. doi: 10.3390/life15060873. PMID: 40566527 PMC12194111

[36] Gavia-García G Rosado-Pérez J Arista-Ugalde TL Aguiñiga-Sánchez I Santiago-Osorio E Mendoza-Núñez VM . Telomere length and oxidative stress and its relation with metabolic syndrome components in the aging. Biology. (2021) 10:253. doi: 10.3390/biology10040253. PMID: 33804844 PMC8063797

[37] Mangge H Herrmann M Almer G Zelzer S Moeller R Horejsi R . Telomere shortening associates with elevated insulin and nuchal fat accumulation. Sci Rep. (2020) 10:6863. doi: 10.1038/s41598-020-63916-6. PMID: 32322021 PMC7176638

[38] Sawicki K Matysiak-Kucharek M Gorczyca-Siudak D Kruszewski M Kurzepa J Kapka-Skrzypczak L . Leukocyte telomere length as a marker of chronic complications in type 2 diabetes patients: a risk assessment study. Int J Mol Sci. (2025) 26:290. doi: 10.3390/ijms26010290. PMID: 39796144 PMC11719939

[39] Liu Y Yue J Jiang Y Tian X Shu A . The role of circrna in insulin resistance and its progression induced by adipose inflammation. J Diabetes Its Complications. (2025) 39:109042. doi: 10.1016/j.jdiacomp.2025.109042. PMID: 40279985

[40] Imamura M Maeda S . Genetic studies of type 2 diabetes, and microvascular complications of diabetes. Diabetol Int. (2024) 15:699–706. doi: 10.1007/s13340-024-00727-4. PMID: 39469559 PMC11512959

[41] Darwish R Alcibahy Y Abu-Sharia G Butler AE . B-cell mitochondrial dysfunction: underlying mechanisms and potential therapeutic strategies. Cells. (2025) 14:1861. doi: 10.3390/cells14231861. PMID: 41369350 PMC12691418

[42] Qin B Wang Y Ding J . The role of mitochondrial function in the pathogenesis of diabetes. Front Endocrinol. (2025) 16:1607641. doi: 10.3389/fendo.2025.1607641. PMID: 40862129 PMC12370513

[43] Yamada Y Kato K Oguri M Horibe H Fujimaki T Yasukochi Y . Identification of four genes as novel susceptibility loci for early–onset type 2 diabetes mellitus, metabolic syndrome, or hyperuricemia. BioMed Rep. (2018) 9:21–36. doi: 10.3892/br.2018.1105. PMID: 29930802 PMC6006760

[44] Ng NHJ Ghosh S Bok CM Ching C Low BSJ Chen JT . Hnf4a and hnf1a exhibit tissue specific target gene regulation in pancreatic beta cells and hepatocytes. Nat Commun. (2024) 15:4288. doi: 10.1038/s41467-024-48647-w. PMID: 38909044 PMC11193738

[45] Cai Y Yao H Sun Z Wang Y Zhao Y Wang Z . Role of nfat in the progression of diabetic atherosclerosis. Front Cardiovasc Med. (2021) 8:635172. doi: 10.3389/fcvm.2021.635172. PMID: 33791348 PMC8006278

