# Supplementary material for: SBSPON gene association with type 2 diabetes mellitus and its impact on leukocyte telomere length
Source: Front Endocrinol (Lausanne). 2026 Apr 16;17:1799481. doi: 10.3389/fendo.2026.1799481 (PMC13128383; doi:10.3389/fendo.2026.1799481)
Supplement: Supplementary file 1 [file DataSheet1.docx]

***SBSPON* gene association with Type 2 Diabetes Mellitus and its impact on Leukocyte Telomere Length.**

Itty Sethi^a^, Gh. Rasool Bhat^b^, Ekta Rai^c^, Swarkar Sharma^d^, Suman Kotwal^e^, Ankit Mahajan^f^, Parvinder Kumar^a,g^ and Manoj K. Dhar^a,f^

Affiliations:

^a^Institute of Human Genetics, University of Jammu, Baba Saheb Ambedkar Road, Tawi, Jammu, Jammu and Kashmir 180006, India

^b^Advanced Centre for Human Genetics, Sher.i.kashmir Institute of Medical Sciences (SKIMS) Soura, J&K, India 180001

^c^School of Life Sciences, Jawaharlal Nehru University, New Delhi, 110067

^d^Department of Zoology, Central University of Jammu, Rahya-Suchani (Bagla), Samba, 181143, Jammu and Kashmir, India

^e^Department of Endocrinology, Government Medical College and Hospital, Jammu

^f^School of Biotechnology, University of Jammu, Baba Saheb Ambedkar Road, Tawi, Jammu, Jammu and Kashmir 180006, India

^g^Department of Zoology, University of Jammu, Baba Saheb Ambedkar Road, Tawi, Jammu, Jammu and Kashmir 180006, India

Corresponding Authors:

Prof. Manoj K Dhar

Institute of Human Genetics and School of Biotechnology

University of Jammu,

Baba Saheb Ambedkar Road, Tawi, Jammu,

Jammu and Kashmir 180006, India

Email: [manojkdhar@rediff.com](mailto:manojkdhar@rediff.com)

Dr. Itty Sethi

Institute of Human Genetics

University of Jammu,

Baba Saheb Ambedkar Road, Tawi, Jammu,

Jammu and Kashmir 180006, India

Email: [bhavya2288@gmail.com](mailto:bhavya2288@gmail.com)

**SUPPLEMENTARY INFORMATION**


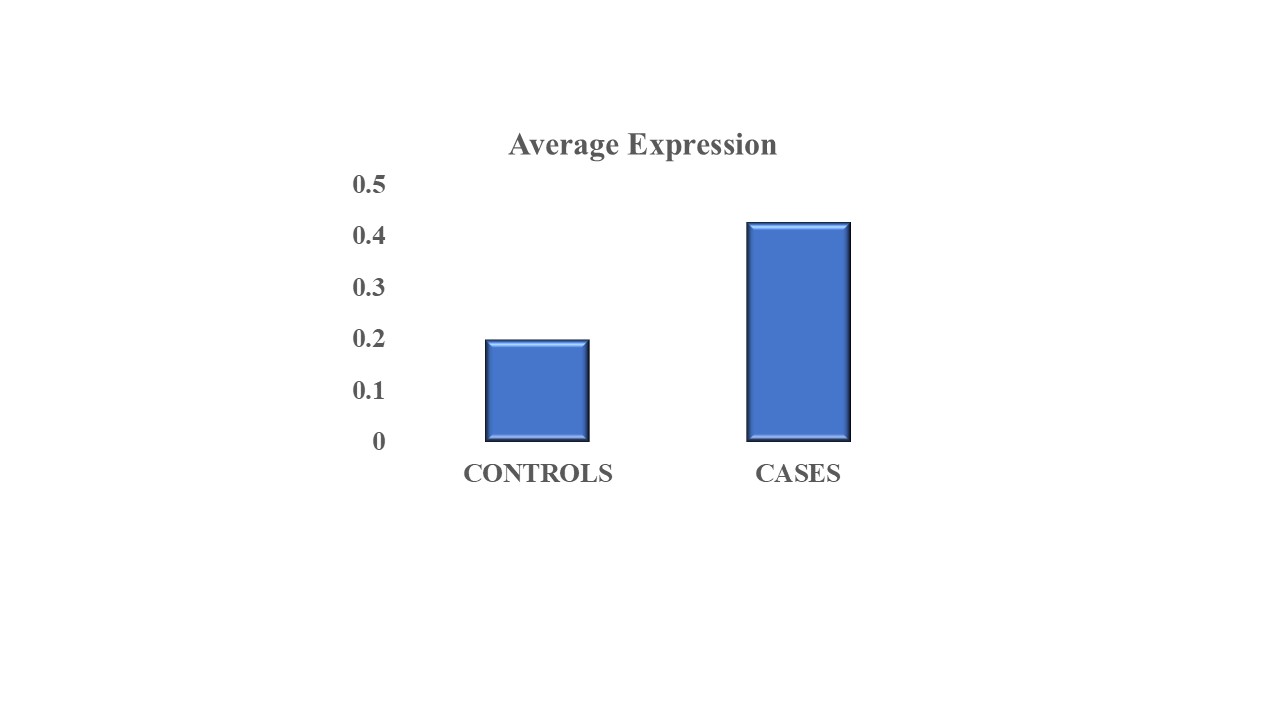


**Supplementary Figure S1: Representation of the expression data.** A significant difference of expression was observed among the cases and controls. Cases showed elevated levels of expression.


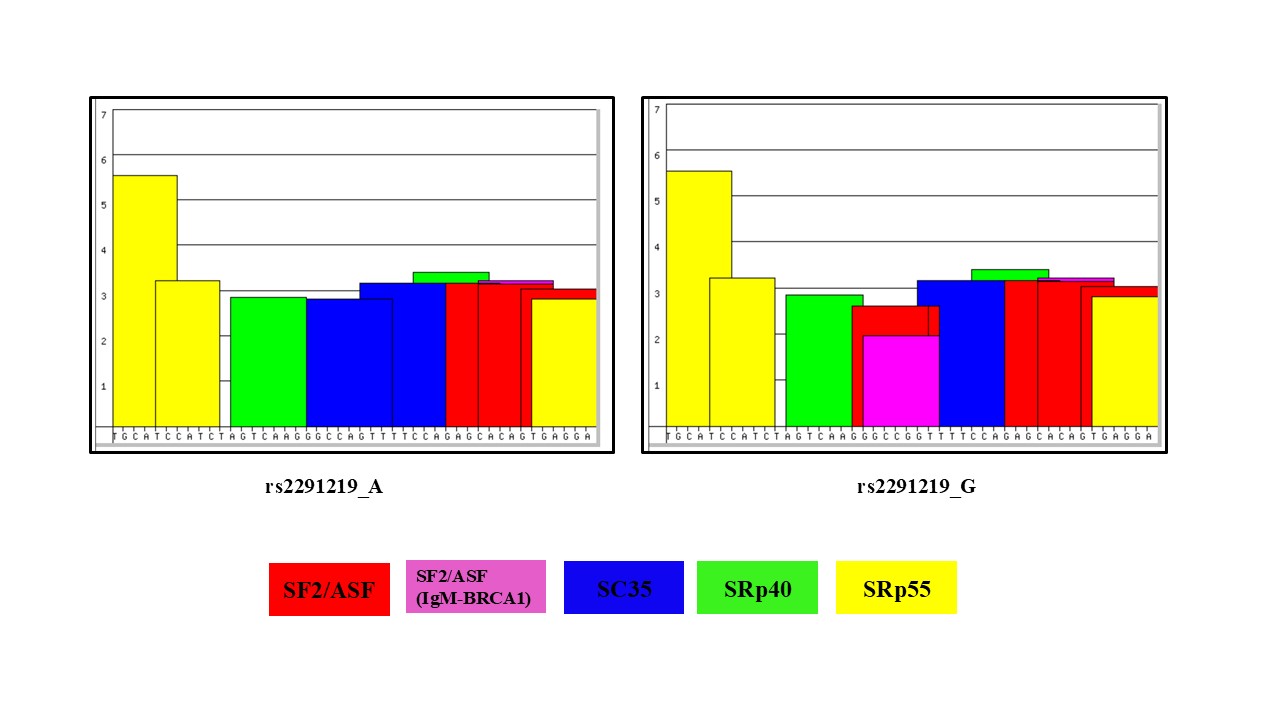


**Supplementary Figure S2: Effect of polymorphism on the Exonic Splicing Enhancers (ESEs) according to ESE prediction tool.** ESE finder enables to identify the potential ESE sites. The height of the colored bars represents the motif scores and the width of the bars indicates the length of the motif. Bars in red, yellow, blue, purple and green indicate potential binding sites for Serine-Arginine (SR) proteins SF2/ASF, SRp55, SC35, SF2/ASF (IgM-BRCA1) and SRp40, respectively. rs2291219_A represents the ESE sequence with the risk allele in the studied population and rs2291219_G represents the ESE sequence with the allele not posing risk in the studied population. From the figure, we can predict that there is a change in the potential ESE sites as can be seen from change in the bars (change in the potential splicing sites) that could lead to the disease susceptibility.


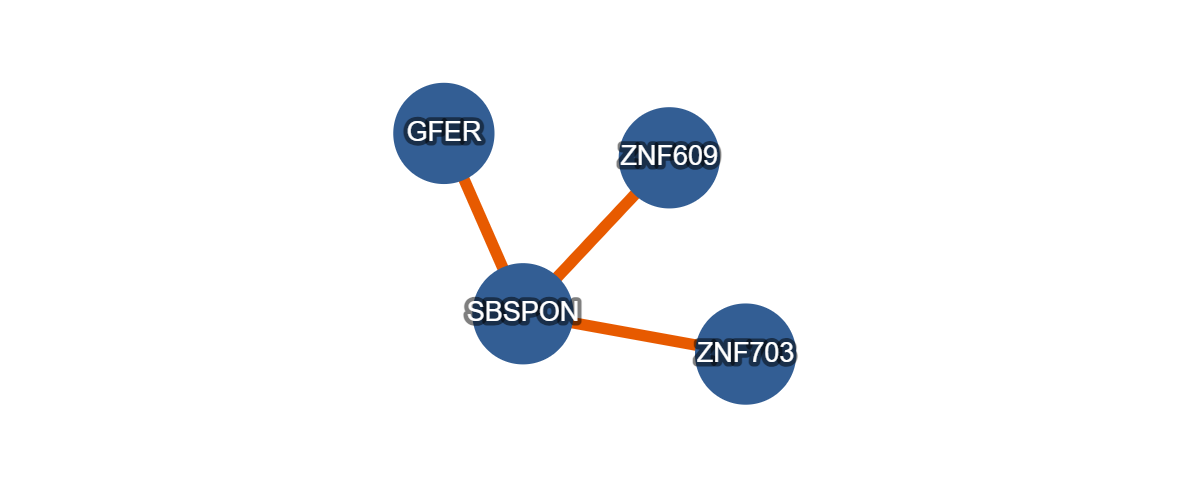


**Supplementary Figure S3: Representation of the EMBL-IntAct database.** The interaction of SBSPON protein with proteins GFER, ZNF609 and ZNF703.


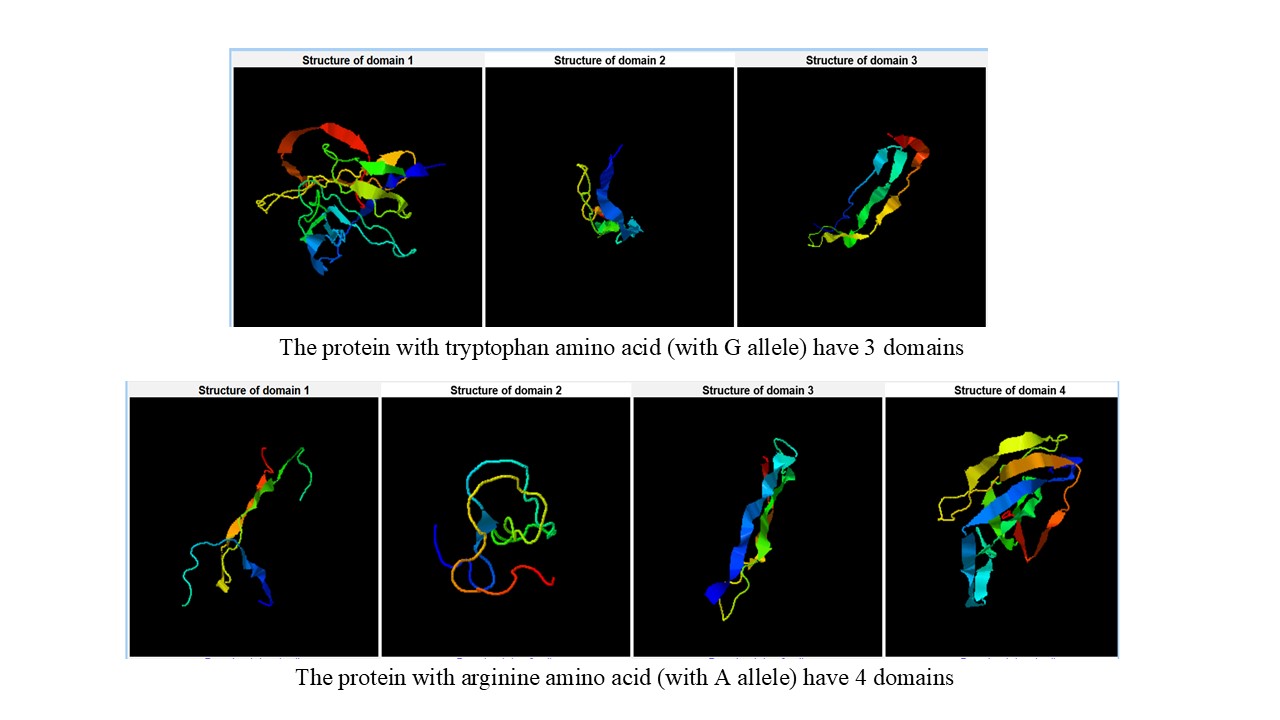


**Supplementary Figure S4: Representation of the difference in protein domains** generated by iTasser MTD.


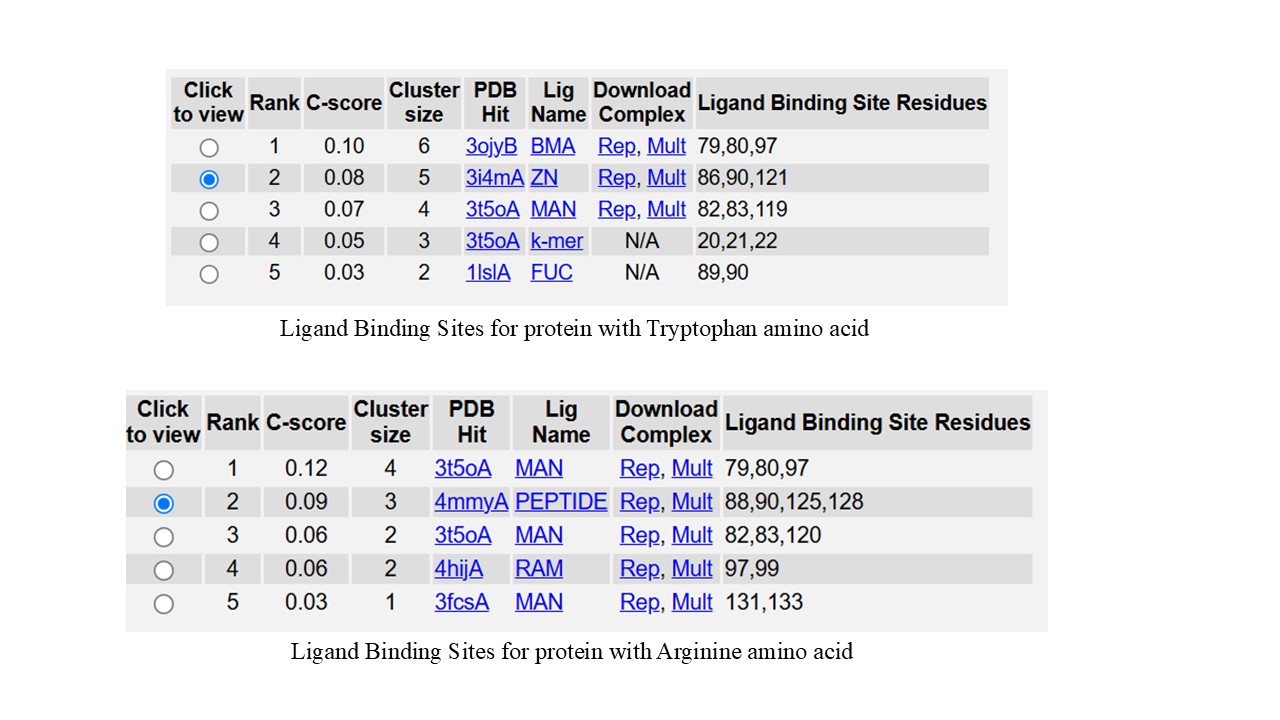


**Supplementary Figure S5: Representation of the ligand binding sites in protein** generated by iTasser software.


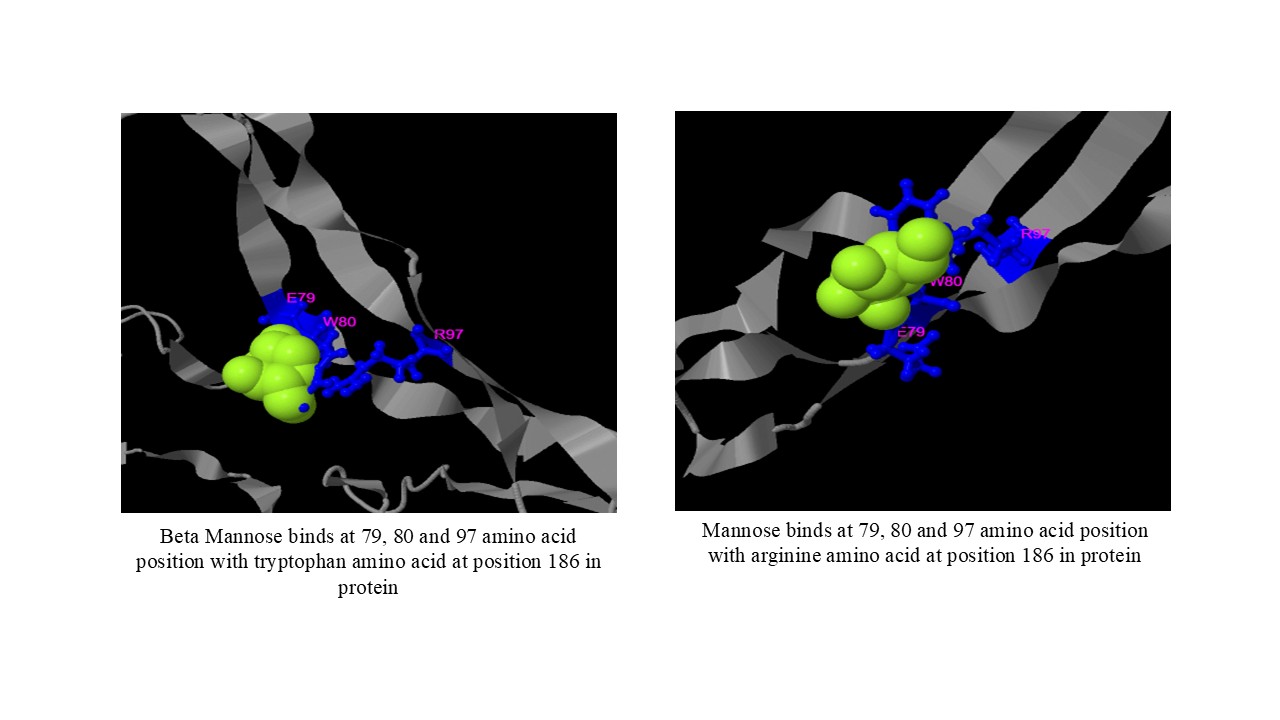


**Supplementary Figure S6: Representation of the ligand binding sites in protein** generated by iTasser softwares**.** Beta mannose binds with protein at position 79, 80 and 97 when the protein contains amino acid tryptophan at position 186 whereas mannose binds at the same positions when arginine is present at position 186 in the protein.


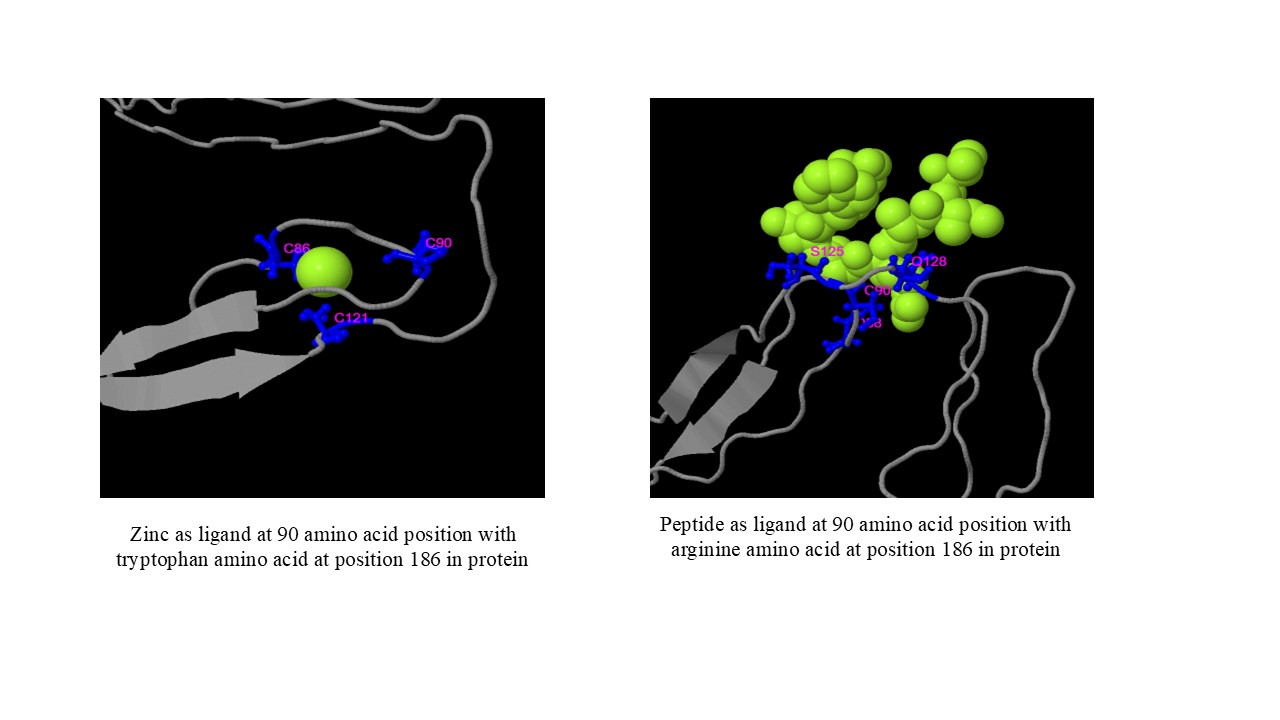


**Supplementary Figure S7: Representation of the ligand binding sites in protein** generated by iTasser softwares**.** Zinc binds with protein at position 90 when the protein contains amino acid tryptophan at position 186 whereas peptide binds at the same positions when arginine is present at position 186 in the protein.


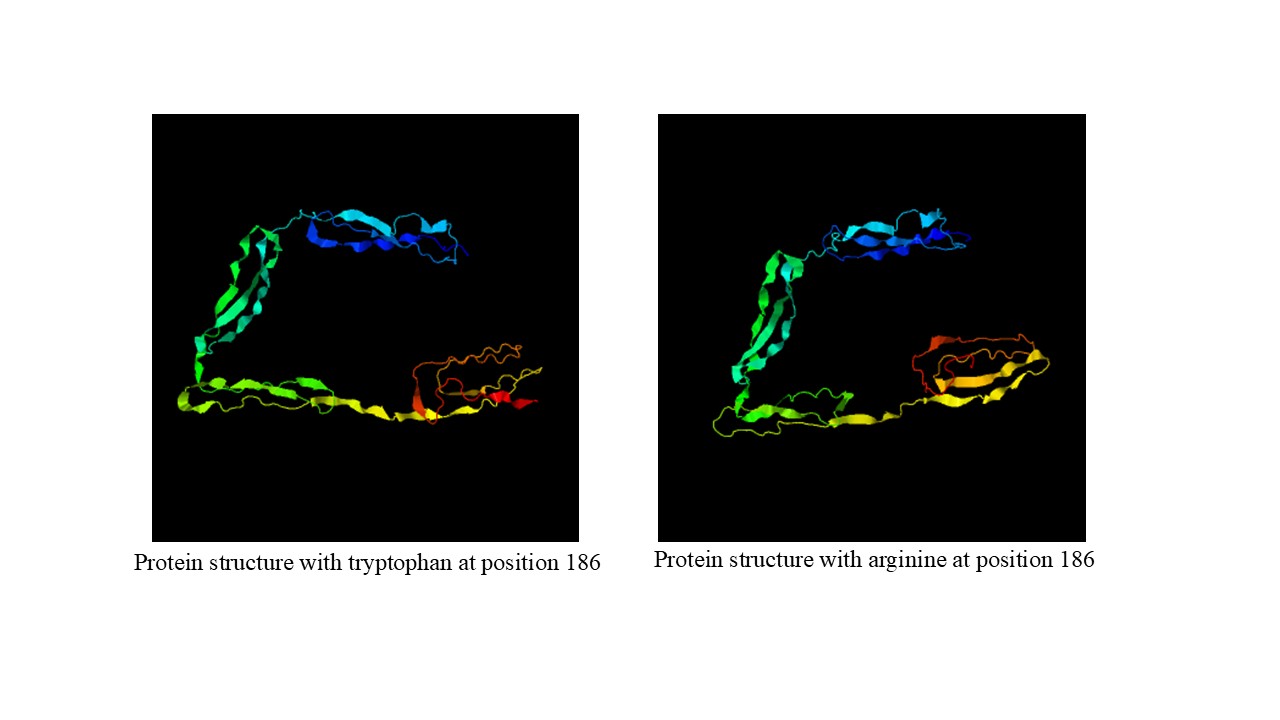


**Supplementary Figure S8: Representation of the Protein structure with tryptophan and arginine amino acid at position 186 respectively.**

**Supplementary Tables**

**Supplementary Table S1:**

| **S.No.** | **Oligo ID** | **Oligo Sequence 5´-3´** |
| --- | --- | --- |
|  | SBSPON_F | TGTTCCTGCCTTTATAACTACCTC |
|  | SBSPON_R | TTTTCCAGAGCACAGTGAGG |
|  | ALB_F (Forward) Primer | AAGCTGAGTTTGCAGAAGTTTC |
|  | ALB_R (Reverse) Primer | ATATCGACGACTCTTTACCCTG |

**Supplementary Table S2:** Correlation analysis of associated variant in the studied population

| **Variant/ Gene** | **rs2291219/ *SBSPON*** | | | | | | |
| --- | --- | --- | --- | --- | --- | --- | --- |
| **Genotype** | **AA** | | **AG** | | | **GG** | |
| **Pearson Correlation in studied population** | Age | Age of Onset of T2DM | | BMI | FBS | | RBS |
|  | -0.057 | -0.11 | | 0.036 | 0.042 | | 0.079 |
| ***P*-value** | 0.09 | **0.04*** | | 0.287 | 0.354 | | 0.065 |
| **FDR adjusted** | 0.15 | 0.15 | | 0.354 | 0.354 | | 0.15 |
| **Pearson Correlation in Males only** | Age | Age of Onset of T2DM | | BMI | FBS | | RBS |
|  | -0.11 | -0.14 | | 0.05 | -0.01 | | 0.05 |
| ***P*-value** | **0.02*** | 0.07 | | 0.23 | 0.84 | | 0.38 |
| **FDR adjusted** | 0.1 | 0.17 | | 0.38 | 0.84 | | 0.47 |
| **Pearson Correlation in Females only** | Age | Age of Onset of T2DM | | BMI | FBS | | RBS |
|  | 0.002 | -0.08 | | 0.015 | 0.104 | | 0.116 |
| ***P*-value** | 0.97 | 0.33 | | 0.77 | 0.12 | | 0.07 |
| **FDR adjusted** | 0.97 | 0.55 | | 0.96 | 0.30 | | 0.35 |

*Correlation is significant at the 0.05 level (2-tailed).

Pearson correlation analysis was performed to evaluate the relationship between the studied variable and clinical parameters. False discovery rate (FDR) correction was applied using the Benjamini–Hochberg method to account for multiple testing. Nominally significant associations (*p* < 0.05) did not remain significant after FDR correction.

**Supplementary Table S3:** Correlation analysis of associated variant in the studied population with respect to the associated genotypic model.

| **Variant/ Gene** | **rs2291219/ *SBSPON*** | | | | | |
| --- | --- | --- | --- | --- | --- | --- |
| **Genotype** | **AA** | | | **AG+GG** | | |
| **Pearson Correlation in studied population** | Age | Age of Onset of T2DM | BMI | | FBS | RBS |
|  | -0.024 | -0.136 | 0.021 | | 0.048 | 0.061 |
| ***P*-value** | 0.5 | **0.02*** | 0.6 | | 0.3 | 0.2 |
| **FDR adjusted** | 0.6 | 0.1 | 0.6 | | 0.5 | 0.5 |
| **Pearson Correlation in Males only** | Age | Age of Onset of T2DM | BMI | | FBS | RBS |
|  | -0.059 | -0.178 | 0.043 | | 0.041 | 0.071 |
| ***P*-value** | 0.2 | **0.02*** | 0.3 | | 0.5 | 0.2 |
| **FDR adjusted** | 0.3 | 0.1 | 0.38 | | 0.5 | 0.33 |
| **Pearson Correlation in Females only** | Age | Age of Onset of T2DM | BMI | | FBS | RBS |
|  | 0.014 | -0.087 | 0.003 | | 0.055 | 0.049 |
| ***P*-value** | 0.8 | 0.3 | 0.9 | | 0.4 | 0.5 |
| **FDR adjusted** | 0.9 | 0.75 | 0.9 | | 0.67 | 0.63 |

*Correlation is significant at the 0.05 level (2-tailed).

**Supplementary Table S4:** One way ANOVA of significant Variant with quantitative traits

| **Variant/ Gene** | **rs2291219/ *SBSPON*** | | | | |
| --- | --- | --- | --- | --- | --- |
|  | **MEAN±SE** | | | ***P*-value** | **FDR adjusted** |
| **Genotype** | **AA** | **AG** | **GG** |  |  |
| **BMI** | 26.05±3.72 | 26.6±5.52 | 26.3±4.45 | 0.5 | 0.69 |
| **Age of onset** | 44.39±9.8 | 47.14±7.9 | 47.13±9.04 | 0.05 | 0.14 |
| **Blood glucose (Fasting) mg/dl** | 156.14±52.14 | 154.7±61.8 | 156.7±56.5 | 0.9 | 0.90 |
| **Blood glucose (Random) mg/dl** | 233.42±75.24 | 225.62±71.8 | 221.76±65.52 | 0.5 | 0.69 |
| **SBP (mmhg) / FDR adjusted** | 134.27±17.05 | 137.36±17.3 | 135.68±18.7 | 0.38 | 0.69 |
| **DBP (mmhg)** | 83.49±6.4 | 83.5±7.01 | 82.91±7.02 | 0.73 | 0.89 |
| **Cholesterol** | 163.82±31.38 | 172.48±38.47 | 180.79±46.4 | **0.01** | **0.037** |
| **Triglycerides** | 206.52±87.9 | 205.04±85.2 | 250.55±185.73 | **0.008** | **0.037** |
| **High Density Lipoprotein (HDL)** | 44.46±7.9 | 46.26±10.5 | 48.57±22.8 | 0.16 | 0.35 |
| **Low Density Lipoprotein (LDL)** | 81.9±26.1 | 83.03±28.1 | 82.5±27.4 | 0.9 | 0.90 |
| **Very Low-Density Lipoprotein (VLDL)** | 40.05±14.4 | 42.2±25.3 | 50.98±38.6 | **0.01** | **0.037** |

**Supplementary Table S5:** One way ANOVA of Variant with quantitative traits with respect to associated genotypic model

| **Variant/ Gene** | **rs2291219/ *SBSPON*** | | | |
| --- | --- | --- | --- | --- |
|  | **MEAN±SE** | | ***P*-value** | **FDR adjusted** |
| **Genotype** | **AA** | **AG+GG** |  |  |
| **BMI** | 26.05±3.72 | 26.53±5.1 | 0.53 | 0.73 |
| **Age of onset** | 44.39±9.8 | 47.14±8.3 | **0.02*** | 0.22 |
| **Blood glucose (Fasting) mg/dl** | 156.14±52.1 | 155.5±59.6 | 0.29 | 0.46 |
| **Blood glucose (Random) mg/dl** | 233.4±75.2 | 224.04±69.2 | 0.15 | 0.41 |
| **SBP (mmhg)** | 134.27±17.1 | 136.69±17.9 | 0.79 | 0.79 |
| **DBP (mmhg)** | 83.49±6.4 | 83.27±7.01 | 0.29 | 0.46 |
| **Cholesterol** | 163.8±31.4 | 175.99±42.1 | 0.09 | 0.33 |
| **Triglycerides** | 206.52±87.9 | 224.29±138.5 | 0.28 | 0.46 |
| **High Density Lipoprotein (HDL)** | 44.5±7.9 | 47.24±16.9 | 0.62 | 0.76 |
| **Low Density Lipoprotein (LDL)** | 81.9±26.1 | 82.8±27.8 | 0.74 | 0.79 |
| **Very Low-Density Lipoprotein (VLDL)** | 40.06±14.4 | 45.9±31.8 | 0.23 | 0.46 |

**Supplementary Table S6:** Binary Logistic Regression analysis of Leukocyte Telomere length among the individuals with T2DM and healthy with all the three genotypes observed in the studied population.

| **Variables** | ***p*-Value** | | |
| --- | --- | --- | --- |
|  | **AA** | **GA** | **GG** |
| **Unadjusted** | 0.01 | 0.03 | 0.35 |
| **Adjusted for** |  |  |  |
| **Age, Gender and BMI** | 0.004 | 0.04 | 0.45 |
| **Age, Gender, BMI, and FBS** | 0.007 | 0.9 | 0.41 |

**Supplementary Table S7:** Correlation Analysis of Telomere length with T2DM parameters.

| **VARIABLES** | **PEARSON CO-EFFICIENT** | ***p*-VALUE** | **FDR-adjusted *p*** |
| --- | --- | --- | --- |
| **Age** | -0.095 | 0.19 | 0.36 |
| **Gender** | 0.17 | **0.02*** | 0.09 |
| **BMI** | -0.09 | 0.2 | 0.36 |
| **Fasting Blood Sugar** | -0.225 | **0.01**** | 0.09 |
| **Random Blood Sugar** | -0.184 | **0.03*** | 0.09 |
| **Cholesterol** | -0.006 | 0.9 | 0.90 |
| **High Density Lipoprotein** | 0.041 | 0.6 | 0.77 |
| **Very Low-Density Lipoprotein** | 0.04 | 0.6 | 0.77 |
| **Triglycerides** | -0.02 | 0.8 | 0.90 |

*. Correlation is significant at the 0.05 level (2-tailed). **. Correlation is significant at the 0.01 level (2-tailed).
